# Effective implementation of the UNCRPD by Thailand State Party: challenges and potential remedies

**DOI:** 10.1186/s12914-017-0123-5

**Published:** 2017-05-25

**Authors:** Donruedee Srisuppaphon, Arnon Sriboonroj, Wachara Riewpaiboon, Viroj Tangcharoensathien

**Affiliations:** 1 International Health Policy Program, MOPH, Bangkok, Thailand; 2 Faculty of Law, Thaksin University, Songkhla, Thailand; 3 Health Systems Research Institute, Nonthaburi, Thailand

**Keywords:** The United Nations Convention on the Rights of Persons with Disabilities, Implementation gaps, State Party report, Alternative report, Disability policy, Thailand

## Abstract

**Background:**

The Thai government ratified the United Nations Convention on the Rights of Persons with Disabilities (UNCRPD) in 2008, and the first progress report by the State Party was issued in 2012. This study assesses and identifies gaps in the Government’s implementation of the Convention.

**Methods:**

Using the Deming Plan-Do-Check-Act Cycle as an analytical framework for continuous quality improvement, we reviewed five documents which are: the 2012 State Party report; the list of issues by the Committee on the Rights of Persons with Disabilities; the 2015 replies to the list of issues by the Thai government; an alternative report produced by Civil Society Organizations (CSOs); and an alternative report produced by the National Human Rights Commission of Thailand. Content analysis is applied to generate the emerging gaps in implementation.

**Results:**

Thailand’s main advantage is the evolving legal frameworks operating in compliance with the convention, although further amendment is still needed, including effective law enforcement. Conflicting information between the Government’s and alternative reports reflects the shortcomings in the information system that intends to support rigorous monitoring and evaluation. Lacking of concrete measures and outcome indicators on certain articles reflects the State Party’s limited understanding of the concept of human rights and participatory approaches and insufficient institutional capacities for effective implementation.

**Conclusions:**

To rectify these implementation gaps, a few actions are suggested. This includes amending the laws which violate the rights of persons with psychosocial disability; reforming governance where the monitoring bodies are truly independent from implementing agencies; strengthening cross-sectoral actions; and improving information systems which facilitate monitoring and evaluation where Disabled People’s Organizations and Civil Society Organizations are recognized as true equal partners. Implementation research can provide evidence for further effective implementation.

## Background

### Disability and the UNCRPD

Disability is not only a biological or medical condition but also a social construction. It needs to be understood as a complex set of personal and environmental requirements for social living [1]. The human values, dignity and rights of persons with disabilities need to be realized as equally as for others. The adoption of the United Nations Convention on the Rights of Persons with Disabilities (UNCRPD) by the United Nation General Assembly on 13 December 2006 marked global recognition of the rights of persons with disabilities. The CRPD as a human rights treaty was opened to signatories on 30th March 2007 and came into force on 3rd May 2008. By 2016, 163 UN member states had ratified it [2].

There are 50 articles in the Convention (See Table 1). Articles one to four describe the purpose, definition, principles and obligations of the States Parties. Articles five to nine are detailed principles on non-discrimination, accessibility, equality between men and women, and respect the right of children with disabilities. Articles 10–30 guarantee the rights of persons with disabilities (PWD). Articles 24–28 entitle basic rights such as education, health and employment. Articles 31–33 guide the country implementation process. Articles 34–50 addresses the process on ratification, government reporting and the cooperation process between States Parties and the Committee on the Rights of Persons with Disabilities (hereafter the Committee) [3, 4].

**Table 1:** Articles of the UNCRPD

Articles in General Concepts	Articles on Rights of PWD		Articles on implementation tools and ratification	
1. Purpose 2. Definitions 3. General Principles 4. General Obligations 5. Equality and Non-discrimination 6. Women with disabilities 7. Children with disabilities 8. Awareness raising 9. Accessibility	10. Right to life 11. Situations at risk and humanitarian emergency 12. Equal recognition before the law 13. Access to justice 14. Liberty and security of the person 15. Freedom from torture or cruel, inhuman or degrading treatment or punishment 16. Freedom from exploitation, violence and abuse 17. Protecting the integrity of the person 18. Liberty of movement and Nationality 19. Living independently and being included in the community	20. Personal mobility 21. Freedom of expression and opinion, and access to information 22. Respect for privacy 23. Respect for home and family 24. Education 25. Health 26. Habilitation and rehabilitation 27. Work and employment 28. Adequate standard of living and social protection 29. Participation in political and public life 30. Participation in cultural life, recreation, leisure and sport	31. Statistics and data collection 32. International cooperation 33. National implementation and monitoring 34. Committee on the rights of PWD 35. Reports by States Parties 36. Consideration of reports 37. Cooperation between States Parties and the Committee 38. Relationship of the Committee with other bodies	39. Report of the Committee 40. Conference of States Parties 41. Depository 42. Signature 43. Consent to be bound 44. Regional integration organization 45. Entry into force 46. Reservations 47. Amendments 48. Denunciation 49. Accessible format 50. Authentic texts

### Disability in Thailand: evolution of perceptions, legislation and responsible agencies

Thailand’s disability movement has been active and dynamic since the 1980s, the decade when the international day of persons with disabilities was announced [5]. Prior to that, persons with disabilities tended to be seen as objects of pity according to their past life (*Karma*). They were called ‘handicapped’ and hidden in their house or institutionalized in welfare centers [6, 7]. This was also reflected by legal attitudes at that time.

*“Beggars, handicapped people, or ill people who are unable to work or do not have families to take care of must be detained at the designated care center.” (Begging Controlled Act, 1941)* [6]

Non-government sectors emerged to respond to the needs of disabled people with the primary aim of providing essential social assistance, health and education [5]. Empowerment activities supported disabled people to get together to increase their visibility in society. This introduced the disabled persons’ peer support movement. The Bangkok Association of the Blind, the first Disabled People’s Organization (DPO) in Thailand, was established in 1967 with support from international volunteers and the Thai Royal Family. Thereafter, many other organizations ‘for’ and ‘of’ PWD were gradually established during the following 20 years [5].

With a strong driving force from PWD leaders and government technocrats, the Rehabilitation for Persons with Disabilities Act, which separated PWDs from beggars, was enacted in 1991 [8, 9]. However, as there were no sanctions, it was call the “merit disability legislation” [10]. Along with the enactment of this law, the first medical rehabilitation center in Thailand was established in the Ministry of Public Health. PWDs were legally defined according to their physical impairments for registration to be entitled to essential medical, educational, vocational and social rehabilitation [11].

Since 1996, a representative of disability leaders has been appointed as a senator to voice their concerns on disability issues. With strong pressure from Thai DPOs and the international disability movement, the Person with Disability Empowerment Act (PDEA) was legislated in 2007 nullifying the 1991 Rehabilitation Act [8, 9].

This new law updated the definition of disability as: *“Persons with limitation in performing activity of daily living or restriction in social participation from personal impairment together with environmental barriers”*. This indicated the major shift towards more social and rights-based concepts of disability in line with the 2006 CRPD [12].

The National Committee for Empowerment of Persons with Disabilities (NCEPD) is chaired by the Prime Minister with members ex-officio who are Permanent Secretaries of concerned Ministries and representatives of DPOs. According to the PDEA, this NCEPD was designated to monitor and enforce implementation of the five-year National Plan on empowering persons with disabilities [9]. The PDEA emphasized that persons with any kinds of disability have dignity and rights, which should be equally respected. At the same time, the 2007 constitution of Thailand and its subordinate legislations endorsed articles on the rights of persons with disabilities as strongly advocated by DPOs [8]. In parallel with PDEA movement, Thai DPO representatives got closely involved with the Ad Hoc Committee in negotiating the CRPD text. Domestic and global movements synergistically supported a policy shift towards the rights of PWD [13]. On July 29th 2008, the government of Thailand ratified the Convention.

Government agencies responsible for law enforcement and implementing the national plan have evolved names and functions in line with the disability laws. Initially in 1940, a small unit functioned under the then Department of Public Welfare, which then upgraded to the Office of the Committee for Rehabilitation of PWD under the 1991 Rehabilitation of PWD Act. In 2007 according to the PDEA, it became the National Office for Empowerment of PWD (NEP) under the Ministry of Social Development and Human Security (MSDHS) acting as the national focal point on disability. The NEP was later restructured and upgraded as the Department of Empowerment of Persons with Disabilities (DEP) with an expanded mandate to integrate and implement policies on disability to enable PWD to access their rights [9].

As mandated by the Convention, States Parties shall submit a comprehensive report to the Committee two years after the entry into force [3]. The Government of Thailand, with some delays, submitted the first report in 2012, which was reviewed by the Committee in 2015 [14]. The Committee produced a list of issues (LOI) [15] on 8 Oct 2015 requesting the State Party to clarify a number of issues. The government had replied to the LOI on 4 Jan 2016 [16] which was reviewed by the Committee in conjunction with two alternative reports produced by the National Human Rights Commission of Thailand (NHRCT) [17] and the Disabilities Thailand (DTH) [18] which is the general council of PWD in Thailand and its network. In March 2016, there was a “constructive dialogue” in Geneva between the Committee and the Thai government on their responses to LOI, with the presence of representatives from NHRCT and DTH. A side event was convened before the dialogue took place between the Committee and representatives from NHRCT and DTH, so that the Committee had a chance to listen to concerns and gaps in the Government’s replies to the LOI and the realities experienced by CSOs on the ground. Two weeks after the dialogue, the Committee issued “concluding observations” and officially transmitted these to the State Party [19, 20].

To understand the CRPD implementation gaps, this study assesses the level of government implementation by reviewing and comparing five reports: the State Party report, the LOI by the Committee, the government’s replies to the LOI and two alternative reports, and identifies the gaps for further improvement. The finding of this study may contribute to other States Parties with similar level of socio-economic context, in their effective implementation and achievement of the goals of CRPD.

## Method

### Document to review

Documents on CRPD implementation are mostly grey literature such as government reports or minutes of meetings; they are scattered across Ministries and are fragmented and difficult to retrieve. The State Party report and the reply to LOI by the government to the Committee were the most reliable and comprehensive sources of information on CRPD implementation. As mandated by the Convention, DTH and NHRCT submitted their alternative reports, which summarized implementation progress during the same period. The whole series of documents submitted to and produced by the CRPD committee during 2012–2016, including the 2012 State Party report [14], the LOI [15], the replies to the LOI by the State Party [16] and the two alternative reports [17, 18], were used as the major sources of information for this review. The relevant documents or those cited by these five documents were retrieved for verification as much as possible.

### Data extraction

**Fig. 1:**
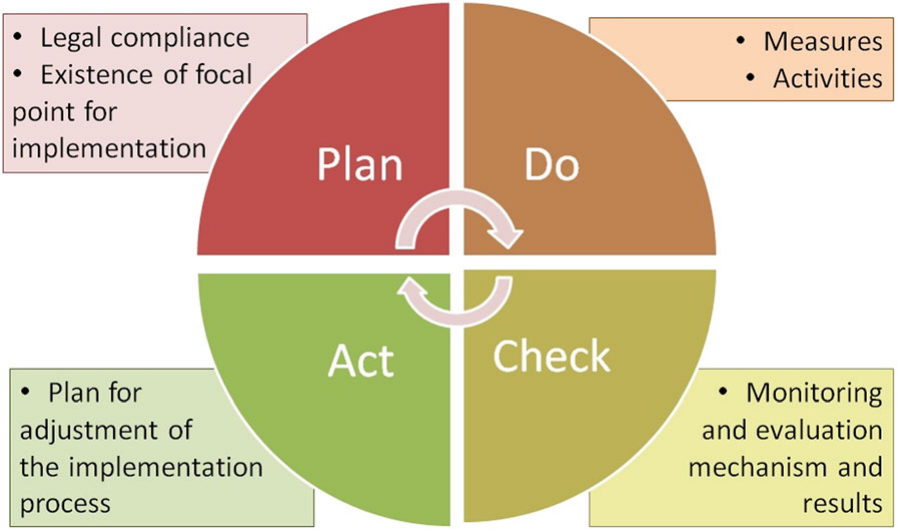
Analysis framework based on Deming ‘PDCA’ Concept

We consider the implementation of the CRPD similar to a process of continuous quality improvement. The Deming concept [21] of Plan-Do-Check-Act (PDCA) was applied to assess the level of implementation in each Article of the CRPD (Fig. 1).

Information from four reports, two from the government and two from alternative reports of DTH and NHRCT, were extracted article by article of the CRPD according to the PDCA framework in which the ‘Plan’ step includes the contextual environment and agencies for implementation, i.e., the legal compliance and clear focal points for implementation. Measures and activities would contribute to the ‘Do’ part. The ‘Check’ part includes the monitoring and evaluation of results against objectives, outcome and impact indicators and barriers. Finally, the ‘Act’ part includes the plan for overcoming barriers and strengthening implementation which is informed by findings from the ‘Check’ stage. The extracted data was categorized by sources of reports. The quality of the information was determined by its usefulness in terms of implementation improvement and collaboration among stakeholders as defined in Table 2. Compliance to the law was determined by the statement in each report as to whether the domestic laws were in line with CRPD concept. If the statements in the reports were unclear, the relevant Acts were further reviewed and verified.

**Table 2:** Criteria for document evaluation

	Do	Check	Act
	Measures/Activities	Monitor & Evaluation	Future plan
-	no information	no information	no information
+	Information available on activities performed but without obvious measures/plans/responsible agencies	Information available on output or process indicators or output/outcome information available only in Reply to LOI	general recommendations/plans
++	Information available on Measures/Plans/Committees or responsible agencies without any activity mentioned	Outcome toward the goal of the Article available (indicating effect to PWD)	Specific recommendations/plans that correspond to findings in the monitor & evaluation
+++	Information available on Measures/Plans and activities by single sector	Outcome toward the goal of the Article available (indicating effect to PWD) with evaluation of implementation barriers	NA
++++	Information available on Measures/Plans and activities by multiple sectors	Outcome toward the goal of the Article available (indicating effect to PWD) with series of monitoring results (indicating KPI monitoring) with evaluation of implementation barriers	NA

Content analysis is applied to generate the emerging implementation gaps. Two researchers independently reviewed the information; where there was disagreement, discussions were made to reach consensus between the two researchers.

## Results

### Overview of the five documents under review

The Thailand State Party report is a 34-page document produced by the government describing what has been implemented for each of the Articles 1 to 33 of the CRPD. The report mostly contained fragmented measures and activities performed by government agencies. Activities performed in collaboration with or solely by CSO or private agencies were reported in some articles.

The list of issues is a four-page document containing one to two questions or statements requested by the Committee for clarification of each Article in relation to the initial report of Thailand. Out of the total 33 Articles reported by the Government, the Committee raised issues in 25 Articles, mostly about the mechanism and measures of implementation, measures and activities for the protection of the rights especially in avoiding substituted decision-making, and the level and channels of participation of PWD.

The replies to the list of issues is a 19-page document submitted by the Thai government in responses to questions or concerns on specific Articles raised by the Committee in its list of issues.

The DTH alternative report is a 68-page document, describing the progression of implementation and identified barriers from PWD’s perspectives. It also summarized the case reports and evidence gathered from PWD assemblies conducted by DPO and CSO, which somewhat disagreed in detail with the State Party report. It also proposed recommendations for each Article in particular four critical issues of concern: the elimination of discrimination against PWD, accessibility, the management of the Fund for Empowerment of PWD and the establishment of Civil Society Organization-based disabilities service center.

The NHRCT alternative report is a 20-page document synthesizing important issues and identifying key missing challenges overlooked by the Government report and proposed practical recommendations in 14 Articles.

From the overview of these five reports, the State Party report seems to focus mainly on the ‘Plan’ and ‘Do’ parts, which reflected incomplete process of implementation, while the ‘Check’ and ‘Act’ parts were mostly raised by the alternative reports. Table 3 shows the summary of finding extracted from these reports. Plenty of disagreements by different reports on legal compliance were demonstrated.

**Table 3:** Finding summary

No	Article of the UNCRPD	Plan		Do	Check		Act	
		Legal compliance		Measures/Activities	Monitor & Evaluation		Future Plan	
		Gov	Alt	Gov	Gov	Alt	Gov	Alt
5	Equality, Non discrimination	C	NC	++	-	+++	-	++
6	WWD	C	NC	++++	+	++	-	+
7	CWD	C	NC	++++	-	++	-	++
8	Awareness raising	C	NS	+	-	+	-	+
9	Accessibility	C	NC	++++	+	+++	-	++
10	Right to life	C	NS	-	-	-	-	-
11	Situation of risk and humanitarian emergency	C	NC	+++	-	++	-	++
12	Equal recognition before the law	C	NC	+++	-	+++	-	++
13	Access to justice	C	NC	+	-	+++	-	++
14	Liberty and security of the person	C	C	+	+	+++	-	++
15	Freedom from torture or cruel, inhuman or degrading treatment or punishment	C	NC	-	-	+++	-	++
16	Freedom from exploitation, violence, abuse	C	C	+++	-	+++	-	++
17	Protecting the integrity of the person	C	NC	-	-	+	-	++
18	Liberty of movement and nationality	C	NC	-	-	+	-	++
19	living independently and being included in the community	C	C	+	+	+++	-	++
20	Personal mobility	C	C	+	+	+++	-	++
21	Freedom of expression and opinion, and access to information	C	NC	++++	++	+++	-	++
22	Respect for privacy	C	NS	-	-	-	-	-
23	Respect for home and family	C	NS	+	-	-	-	-
24	Education	C	C	+	++	+++	-	++
25	Health	C	NS	+	+	+++	-	++
26	Habilitation and rehabilitation	NS	NS	+	+	+++	-	++
27	Work and employment	C	C	++++	++	+++	-	++
28	Adequate standard of living and social protection	C	C	+++	+	+++	-	++
29	Participation in political and public life	NC	NC	+	++	++	-	+
30	Participation in cultural life, recreation, leisure, sport	NS	C	+	-	+	-	++
31	Statistics and data collection	NA	NA	+	+	++	+	++
32	International cooperation	NA	NA	+	-	-	-	-
33	National implementation and monitoring	NA	NA	+	-	+++	-	++

*Abbreviation: C* comply, *NC* not comply, *NS* not stated, *NA* not applicable

### Legislative compliance with CRPD: gaps remain

Although the State Party report confirmed legal compliance to almost all CRPD Articles, alternative reports provided evidence that several Articles in the PDEA 2007 were not fully compliant with the CRPD. For example, discriminatory actions for academic purposes or public interest are still allowed, and denial of reasonable accommodation^1^ is still not considered as discriminatory practice against PWD. In addition, the PDEA 2007 still neglects to adhere to the quota of women with disabilities serving as members of the National Committee for Empowerment of PWD (NCEPD), while the quota regarding types of disability was explicitly determined. Additionally, the rights of children with disabilities were not specifically mentioned anywhere.

Moreover, some Articles in certain laws obviously do not comply with the CRPD, for example, the Civil and Commercial Code which prohibits deaf and blind people from being witnesses in the inheritance process and the 2008 Mental Health Act allows forced treatment and institutionalization of mentally ill patients. Most importantly, serious concerns were raised by the DTH and the CRPD committee that the 2016 draft Constitution fails to maintain the text that specified the rights of PWD as it was in the previous Constitution. For example, it revokes the rights of PWD in accessing the public environment.

### Implementing and monitoring bodies

With reference to Article 33 of the State Party report (Para 185, 186, 189 Page 33–34), three main bodies are responsible for the implementation and monitoring process under the NCEPD, which is the national government authority on enforcing the PDEA and CRPD. Firstly, the Department of Empowerment of Persons with Disabilities (DEP) under the Ministry of Social Development and Human Security (MSDHS) is the national focal point for implementation and self-monitoring process. Secondly, the Sub-committee for the Convention appointed by the NCEPD, chaired by the Permanent Secretary of MSDHS and with the DEP as a technical secretariat is the specific monitoring body. Third, the National Human Right Commission of Thailand (NHRCT) serves as an independent monitoring body. Some other Ministries are also implementers for certain issues, for example, the Department of Disaster Prevention, the Ministry of Interior for Article 11 (Humanitarian emergencies), the Ministry of Justice for Article 13 (Access to justice), the Ministry of Education for Article 24 (Education), the Ministry of Public Health for Article 25 (Health), and the Ministry of Labor for Article 27 (Work and employment).

The DTH alternative report (para 136, page 65) raised concerns about the dual role of the DEP, which acted on both implementing and specific monitoring functions. It noticed that the DEP acted more on the implementer role than on the monitoring and evaluation role. The NHRCT alternative report pointed out that the Sub-committee for the convention was practically set up to prepare the Government report to be submitted to the CRPD Committee. There was no evidence to reflect its function on monitoring, evaluating or addressing problems related to the implementation of the Convention on a regular basis.

### Implementation measures and activities

The government reported on Article 10 (Right to life), 15 (Freedom from torture or cruel, inhuman or degrading treatment or punishment), 17 (Protecting the integrity of the person), 18 (Liberty of movement and nationality), and 22 (Respect for privacy) noting that the related domestic laws and regulations had complied with CRPD; this was done without evidence of other important measures or activities in detail to substantiate its statements. In about half of these Articles, 14 out of 29, a number of scattered activities reported without clear evidence what interventions or measures were applied to achieve the goal of each Article.

There are five articles where National Plans are in place. Issues relating to Article 5 (Equality and non-discrimination), Article 6 (Women with disabilities) and Article 9 (Accessibility) were included in the National Plan of Empowerment of PWD. The essence of Article 11 (Situations of risk and humanitarian emergencies) was covered by the National Plan for Disaster Prevention and Mitigation developed in line with Sendai Framework for disaster risk reduction. Some issues relating to Article 16 (Freedom from exploitation, violence and abuse) were covered by the National Plan on Prevention, Suppression and Remedy of Domestic and Transnational Trafficking in Children and Women but it failed to address specific measures and activities on PWD. Despite the existence of a plan of action, no clear key performance indicators for monitoring the progress of CRPD implementation was stated.

Multiple Acts, subordinate legislations and ministerial regulations, policies, activities were reported in Article 7 (Children with Disabilities or CWD); however, most of them highlighted interventions for children in general with no specific measures to guarantee rights of CWD.

Articles 12, 14, 15, 16, 17, 29 contain critical issues on human rights such as substituted decision making, right to vote, institutionalization, forced treatment and sterilization, confinement and restraint in mental health facilities for persons with psychosocial, behavioral, mental and intellectual disabilities. With reference to these articles, the Government did not reply clearly to questions on specific measures used as requested in the LOI. Some of the replies on measures against forced sterilization (Para 50 in the replies to the LOI) and institutionalization in Half Way Home (Para 40 in the replies to the LOI), clearly contradicted the alternative report (Para 74–75 and Para 63 in DTH alternative report respectively).

### Monitoring and evaluation

Two out of three main monitoring bodies, as reported by the State Party, are Government agencies. Despite their existence, the outcome and impact of implementation in each Article was scarcely described in the State Party report. Yet some of the reported outcomes were in conflict with the alternative reports.

Among five articles with the National Plan supporting implementation, ‘Accessibility’ is the only Article in which six output indicators were reported, such as having supportive laws, academic training courses, and package of knowledge on universal design. However, there were no outcome or impact indicators on improved physical environment, transportation and information accessibility (Para 34, State Party report).

The government was able to demonstrate some results in 13 out of 33 Articles which required reporting to the CRPD Committee. Of these, seven reported on the process of implementation or demonstrated some preliminary outputs which mostly were not the same outputs as monitored and described in the alternative reports. Six Articles (14, 21, 24, 27, 29, 31) reported some outcomes. Of these six articles, the results of Article 14 ‘Liberty and Security of the Person’ and Article 31 ‘Statistics and Data Collection’ were very superficially reported in the replies to the LOI, concerning the number of PWD being institutionalized and the estimated number of PWD.

*‘At present, approximately 500 persons with disabilities who stay in the Government institutions are those who have no families or are abandoned by their families’* (Para 41, in the replies to the LOI of Article 14)

*‘In previous disability surveys done by the National Statistical Office, the number of persons with disabilities was lower than the WHO’s estimates due to different criteria and questions asked in the survey’* (Para 66, in the replies to the LOI of Article 31)

In the remaining four Articles - Article 21 (Freedom of Expression and Opinion, and Access to Information), Article 24 (Education), Article 27 (Work and Employment) and Article 29 (Participation in Political and Public Life) - the State Party report contained the richest information regarding results; however, the results were in conflict with the alternative report.

For example, the Government report referred to statistics from the Ministry of Science and Technology, and demonstrated that the total 810 websites were accessible by PWD (Para 91, State Party report); while the alternative report referred to the Ministry of Information and Communication Technology (ICT) and the Association of the Blind’s survey that none of 64 samples websites, of which 44 were governmental, had passed the World Wide Web Consortium (W3C) standard for disability access [22] (Para 103, DTH alternative report).

Also, the government, with reference to National Statistical Office (NSO) 2007 survey, reported that approximately 450,000 PWDs were uneducated (Para 102, State Party report); this contradicted with almost double the number of around 750,000 uneducated PWD in the alternative report which was based on the DEP’s 2015 report (Para 104, DTH alternative report). In addition, the government replied to the LOI (Para 58, Replies to LOI) that around 360,000 PWD were employed according to NSO’s 2012 survey, while the alternative report referred to DEP’s 2015 report that only approximately 250,000 PWD were employed (Para 121, DTH alternative report).

While most of the government report describes what has been implemented, only two Articles identified implementation challenges. Article 24 ‘Education’ raises the problem that “*In practice schools in some instances continue to be unwilling to accept students with disabilities.*” (Para 123) [14]. In Article 29 on ‘Participation in political and public life’, the challenges are *“Thailand has enacted some laws which limit the political rights for classes of disability including mental, behavioral and autistic.”*(Para 172) [14]. Despite these challenges, the Government did not identify or suggest policy measures or actions for future improvements in their replies to the LOI.

Compared with the State Party report and replies to LOI, the two alternative reports demonstrated both qualitative and quantitative evidence and a number of case studies identified at the grassroots level, which highlighted certain shortcomings with proposed concrete and constructive recommendations in almost all Articles.

## Discussion

The strength of the Thai government on implementing CRPD lies in three main areas including a) early ratification of the CRPD reflecting national commitment to PWD; b) the progressive development of legal frameworks and subsequent amendments in line with CRPD and; c) the regular national household survey in which the disability definition has been evolving to be harmonized with the International Classification of Functioning, Disability and Health (ICF) and CRPD. These gradual changes provide a conducive environment for CRPD implementation.

However, analysis of the State Party report, the replies to the LOI, and the two alternative reports reflect certain shortcomings such as the information system lacking support for quality monitoring and evaluation, inadequate multi-sectoral participation and genuine engagement of DPO and CSO, and low-level internalization of CRPD concept by the implementers particularly among Government officers.

Challenges in monitoring and evaluation are evident in several aspects. For example, the lack of reports on outcomes set against specific indicators of many Articles in the State Party report. Many of the results mentioned by the Government were mainly relating to output and process, for example the key performance indicators of National Plan on Accessibility (Article 9, Para 34 in State Party report). This reflected what had been conducted by the Government agencies but not on the outcome of whether PWD could access public space and transportation.

The information provided in the State Party report is fragmented and unorganized; as a consequence, it cannot demonstrate systemic performance on CRPD implementation. There are very few Articles for which the Government was able to report outcomes. Some outcomes, for example, the number of institutionalized PWD (Article 14) and statistics of PWD (Article 31), were not reported in the 2012 State Party report. They vaguely appeared in the replies to the LOI, reflecting the unavailability of specific predetermined monitoring indicators of each Article. Moreover, the conflicting statistics between the Government and alternative reports (Article 21, 24, 28) reflect the lack of intersectoral collaboration and communication and involvement by DPO to establish common targets. This can consequently create problems in data integration and production of information among stakeholders.

As reflected in the State Party report, the output such as number of activities, meetings and workshops rather than outcome- and impact-oriented implementation reflected the lack of concern about program effectiveness, where these outputs translate into tangible outcomes and impact. As discussed in Discourses of the Thai State on Development during 1961–1996 [23], it was obvious that the Governments’ development programs were predominantly influenced by westernization and modernization theories. In this regard, the State commonly determined what should be done about a particular development agenda rather than focusing on citizen or beneficiary values. With this state-centered concept [24], citizen and community are considered an empty vessel, and it is assumed that any action of the State would lead to successful development programs [25].

Another barrier towards CRPD implementation is the ineffective intersectoral actions across ministerial agencies, as well as inadequate participation by other private and non-state actors. This is evident in Articles about general concepts such as women with disabilities (Article 6) and children with disabilities (Article 7) which are the subjects that need collaboration from multiple government and non-government sectors. However, the State Party report showed the involvement of only one to two line ministries responsible for each service without concrete results. Moreover, large amounts of funding were spread over different fragmented program activities without clear indicators on the outcomes and impact on CWD. Intersectoral actions are critical to solve complex societal challenges [26, 27]. The MSDHS as the focal Ministry has yet to build a shared vision towards PWD where different Ministries feel the benefit of ‘working together’ through the shared vision to achieve their own institutional mandates and goals.

The lack of concrete measures and key performance indicators for monitoring and evaluation in spite of numerous National Plans raises questions about the institutional capacities of DEP and the Sub-committee in coordinating cross-Ministerial, cross-sectoral actions. This might be a result of the deep-rooted “silo” nature of the bureaucratic system which is a major factor prohibiting effective development [28].

Another problem worth noting is the DEP’s dual roles of implementation and monitoring and evaluation functions under the MSDHS. Such conflicted roles might create bias in evaluation, satisfying only on their performed activities instead of considering the most appropriate performance indicators. An independent monitoring committee in which members are balanced between government officers, PWD representatives and disability technocrats, under the NCEPD is highly recommended.

Internalization of CRPD principles across Ministerial Government officials and translation of the principles into their conduct of business is essential but still very limited. The different point of views on ‘legal compliance to CRPD’ between the State and alternative reports also reflect the problem of the Government’s understanding in applying CRPD. One good example is the case of defining the ‘denial of reasonable accommodation’ in the PDEA whether it is considered as discriminatory practice or not. Others are the prohibition of the deaf and the blind from being witness in the inheritance process in the Civil and Commercial Code and the allowance of forced treatment and institutionalization of mentally ill patients in the Mental Health Act.

Furthermore, the medical model of disability, evidently prevailing in several chapters of the Government report, may oppose the individual autonomy and increase the risk of violating PWD’s human rights. For example, the forced treatment by medical professionals, the disability prevention measures by the Ministry of Public Health (Para 130, Article 25 of State Party report), and disability registration which still uses impairment criteria assessed by physician without social participation assessment.

Participation of PWD in all decision-making is another crucial concept of CRPD. However, the capacity of DPOs in driving CRPD learning processes is also questionable. Many PWD representatives are involved in a number of CRPD implementation mechanisms, yet the understanding of CRPD concept is still not applied effectively, especially in cases of human rights denials.

With regards to the little amount of outcome data in the Government report (Articles 21, 24, 27, 29), they are still promising as they are comparable to the international benchmark as recommended by the Incheon strategy. For example, the number of students with disabilities enrolled in school, the number of PWD employed, and the number of accessible websites [29]. However they do not reflect the percentage of coverage due to the lack of a denominator for each indicator. Data on participation in political events is almost comparable to the Danish Gold Indicator in CRPD monitoring [30]. Nonetheless, it is worth noting that these Articles are related to welfare and general assistance to protect human rights. The dominant achievement in these Articles compared to the low level of implementation in the key Articles on the general concept of CRPD such as Equality and Non-discrimination, and Women and Children with Disabilities also raise concerns regarding the internalization of CRPD in Thailand.

It is obvious that Thailand’s legal and policy contexts, as well as the existence of key government agencies, pave the way toward CRPD implementation. The biggest barriers are the inadequate understanding and internalization of the CRPD concept, which comes in parallel with limited participatory approaches. This double obstacle could falsely direct the course of implementation from the ‘plan’ to ‘do’ stage in the Deming cycle. The DEP’s institutional conflicted role and its inadequate institutional capacity on cross-sectoral and cross-Ministerial management further hampers the implementation and in establishing common indicators for M&E. As a consequence the ‘check’ and ‘act’ remain the weakest links in the cycle. Lastly, the inadequate capacities of DPO/CSOs and the lack of their participation and fragmented yet conflicting information systems worsen monitoring and evaluation. Objective robust evidence from M&E for program re-orientation toward a human rights approach is inevitably needed.

**Fig. 2:**
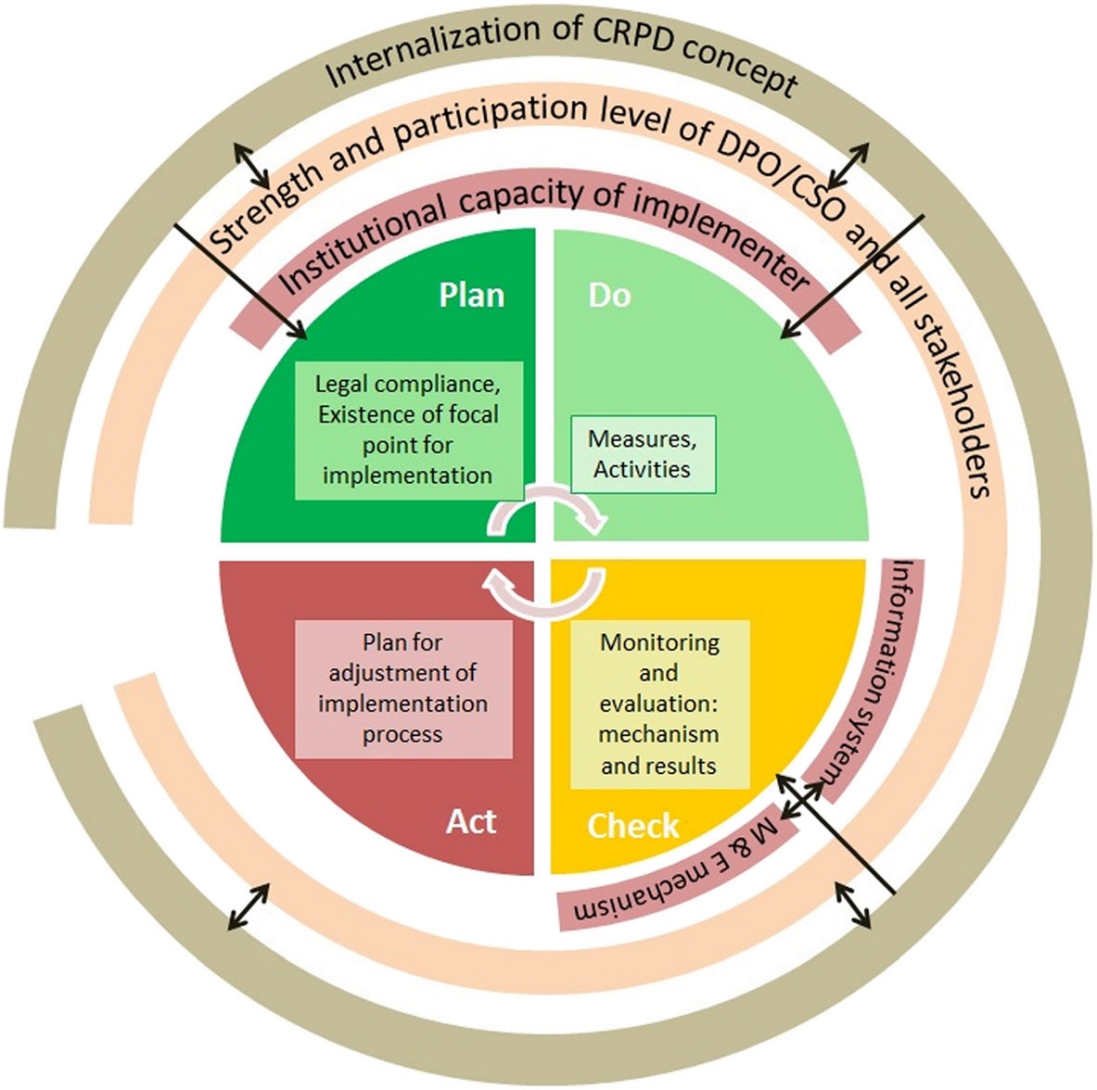
Framework identifying factors affecting the implementation process

Figure 2 depicts the Deming cycle of continuous development; the three outer rings are factors identified as barriers that hinder Thailand’s effective CRPD implementation. The plan for adjustment of the implementation process in the ‘act’ is grossly lacking in Government reports, while many recommendations were stated in the two alternative reports.

There are certain limitations in this study mostly due to the inability to extensively retrieve references of necessary documents. The verification of information relies mostly on triangulation of information from three main sources, namely the State Party reports, the DTH report and the NHRCT report.

The non-compliance of national law to each Article is primarily from argument raised by the DTH report. Although some studies reviewed the Thai legal framework [8, 10], more in-depth exploration to provide specific recommendations for amendment requires further study. Due to the lack of information and unstructured nature of the report, it is difficult to verify the lead actors or implementers in each Article and the collaborative mechanisms among the relevant stakeholders. It should also be noted that the results regarding information from alternative reports are mixed between DTH and NHRCT reports; however, in practice, these two agencies do not conjunctively monitor the Convention. Besides, the information retrieved from five main documents is insufficient to extensively criticize the strength and weakness of DTH and NHRCT. Recommendations to strengthen the capacity of these external monitoring and evaluation bodies need to be adjusted towards the internal context of each agency.

## Conclusion

Thailand is committed to a rights-based disability policy where legal compliance to CRPD is a pivotal entry point for inclusive development, enshrined in the Sustainable Development Goals which aim to “leave no one behind”. The CRPD fosters human right protection of all PWD. The effectiveness of implementation depends on the true understanding and internalizing of the principles of CRPD by Government officials as well as other cross-sector actors. Specific indicators, independent monitoring bodies and regular reporting are key instruments in holding Government actors accountable.

To protect the rights of PWD, the Thai Government should have zero tolerance to the human rights violation of persons with psychosocial disability in Article 12, 14, 15, 16, 17, by amendment of relevant laws which are still incongruent with the CRPD, in particular the 2008 Mental Health Act.

It is strongly recommended to revise the governing structure where the monitoring bodies are truly independent from the implementing body to avoid institutional role conflicts and strengthen cross-sectoral collaboration and monitoring and evaluation mechanisms for which DPO and CSO are true participants not subordinates to DEP or MSDHS. Monitoring and evaluation needs significant improvement, while implementation research to understand policy implementation gaps [31, 32] is required to provide evidence for further development in the ‘Act’ stage of the cycle.

## Abbreviation

CSO: Civil society organization
CWD: Children with disabilities
DEP: The Department of Empowerment of Persons with Disabilities
DPO: Disabled People’s Organizations
DTH: The Disabilities Thailand
FEPD: The Fund for Empowerment of Persons with Disability
ICF: International Classification of Functioning, Disability and Health
ICT: Information and Communication Technology
LOI: List of issues
MSDHS: The Ministry of Social Development and Human Security
NCEPD: The National Committee for Empowerment of Persons with Disabilities
NHRCT: The National Human Rights Commission of Thailand
NSO: The National Statistical Office
PDEA: Person with Disability Empowerment Act
PWD: Person with disability
SEDPD: The Sub-Committee on Elimination of Unfair Discrimination against PWD
UNCRPD: The United Nations Convention on the Rights of Persons with Disabilities; CRPD is used in text for short
W3C: The World Wide Web Consortium
WWD: Women with disabilities

## Footnotes

1
**Reasonable accommodation** means necessary and appropriate modification and adjustments not imposing a disproportionate or undue burden, where needed in a particular case, to ensure to persons with disabilities the enjoyment or exercise on an equal basis with others of all human rights and fundamental freedoms [2]_._
